# Novel thymoquinone lipidic core nanocapsules with anisamide-polymethacrylate shell for colon cancer cells overexpressing sigma receptors

**DOI:** 10.1038/s41598-020-67748-2

**Published:** 2020-07-03

**Authors:** Lydia Ramzy, Abdelkader A. Metwally, Maha Nasr, Gehanne A. S. Awad

**Affiliations:** 1 Department of Pharmaceutics and Industrial Pharmacy, Faculty of Pharmacy, Ain Shams University, Monazamet El Wehda El Afrikia St., El Abbassia, Cairo, 11566 Egypt; 2 Department of Pharmaceutics, Faculty of Pharmacy, Health Sciences Center, Kuwait University, Kuwait City, Kuwait

**Keywords:** Cancer therapy, Targeted therapies, Nanoparticles

## Abstract

The biggest challenge in colorectal cancer therapy is to avoid intestinal drug absorption before reaching the colon, while focusing on tumor specific delivery with high local concentration and minimal toxicity. In our work, thymoquinone (TQ)-loaded polymeric nanocapsules were prepared using the nanoprecipitation technique using Eudragit S100 as polymeric shell. Conjugation of anisamide as a targeting ligand for sigma receptors overexpressed by colon cancer cells to Eudragit S100 was carried out via carbodiimide coupling reaction, and was confirmed by thin layer chromatography and ^1^H-NMR. TQ nanocapsules were characterized for particle size, surface morphology, zeta potential, entrapment efficiency % (EE%), in vitro drug release and physical stability. A cytotoxicity study on three colon cancer cell lines (HT-29, HCT-116, Caco-2) was performed. Results revealed that the polymeric nanocapsules were successfully prepared, and the in vitro characterization showed a suitable size, zeta potential, EE% and physical stability. TQ exhibited a delayed release pattern from the nanocapsules in vitro. Anisamide-targeted TQ nanocapsules showed higher cytotoxicity against HT-29 cells overexpressing sigma receptors compared to their non-targeted counterparts and free TQ after incubation for 48 h, hence delineating anisamide as a promising ligand for active colon cancer targeting.

## Introduction

Sigma receptors are expressed in normal cells of various organs including brain, kidney, liver, immune, endocrine and reproductive organ^1,2^. Two distinguished subtypes were recognized and known as sigma-1 and sigma-2^3^. Neurosteroids are the likely endogenous ligands for sigma-1 receptors^4^, and *N*,*N*-dimethyltryptamine; a natural alkaloid, was also found to be an endogenous ligand for sigma-1 receptors^5,6^. A correlation between sigma receptors and diseases such as Parkinson’s, Alzheimer’s disease and cancer has been proposed^7^, and ligands for sigma-1 receptor were proven to improve various neurodegenerative disorders^8^. Sigma-2 receptor is thought to be crucial for cell morphology, survival and differentiation^9^. Sigma receptors are overexpressed in cancer cells of various organs^10^ including cancers of the brain^11^, lung^12,13^, breast^14^, prostate gland^15^, kidney and colon^16^. The tumor environment characterized by oxidative stress, hypoxia, low pH and insufficient supply of glucose and amino acids mediates disruption of endoplasmic reticulum protein folding. This in turn contributes to sigma-1 receptors activation to promote cell survival^7,17^. In addition, sigma-2 receptors upregulation in cancerous tissues compared to healthy ones has been widely demonstrated^18,19^.

Benzamide derivatives demonstrate a high affinity towards sigma receptors^20–22^. Anisamide (AA); a small molecular weight benzamide derivative was proven to exhibit high affinity for sigma receptors^23–25^. In a review article previously published by our team the use of AA as a targeting ligand in several drug delivery systems was detailed^26^. AA-functionalized doxorubicin-loaded liposomes caused higher tumor growth suppression compared to non-targeted ones in mice bearing the sigma receptor-overexpressing tumor; DU-145 prostate adenocarcinoma. In addition, AA-targeted liposome-polycation-DNA nanoparticles^27^ demonstrated higher cellular uptake by the sigma receptor-overexpressing B16F10 murine melanoma cells than their non-targeted counterparts. Furthermore, lipid/calcium/phosphate nanoparticles^28^ functionalized with AA reduced metastatic nodules in the lung of B16F10 tumor-bearing mice. Similarly, AA-targeted PEGylated lipid/calcium/phosphate nanoparticles loaded with hypoxia-inducible factor 1 alpha (HIF1α) siRNA coupled with photodynamic therapy reduced tumor size in nude mice bearing oral squamous cancer cells with sigma receptor expression^29^. Moreover, in vivo studies indicated that AA-conjugated liposomal calcium carbonate nanoparticles^30^ exhibited higher uptake of a therapeutic peptide by cancerous cells compared to non-targeted nanoparticles, in mice bearing H460 sigma receptor-expressing non-small cell lung carcinoma. In the two latter studies, the targeted nanoparticles highly accumulated in tumors, and were hardly detected in healthy organs^29,30^. In vivo biodistribution studies in A549 lung cancer-bearing mice revealed higher tumor accumulation of gemcitabine delivered by AA-targeted PEGylated chitosan nanoparticles^31^ compared to other organs. AA-targeted cyclodextrin nanoparticles were proven to be a promising delivery system for siRNA in prostate cancer^32,33^. Fitzgerald et al. reported that AA-targeted cyclodextrin nanoformulation caused higher gene knockdown in prostate cancer cells than the untargeted system^32^. A later study highlighted the higher gene silencing displayed by AA-targeted cyclodextrin nanoparticles compared to the non-targeted counterparts in prostate cancer cells grown in a 3D bone metastasis model^33^. In addition, the higher accumulation of AA-conjugated nano-liquid crystalline nanoparticles in MDA-MB 231 breast cancer cells than the non-targeted counterpart was attributed to AA binding to sigma receptors highly expressed by this cell line^34^. Moreover, the intravenous injection of AA-targeted gold nanoparticles loaded with siRNA combined with intraperitoneal paclitaxel injection resulted in an enhanced antitumor activity in prostate cancer PC3 xenograft mouse model compared to using either one of the two therapies individually^35^.

In our research, Eudragit S100 polymeric nanocapsules were prepared for targeting colon cancer. Eudragit S100; one of the pH-dependent polymers used in oral drug delivery^36^ is based on methacrylic acid and methylmethacrylate units in the ratio 1:2. The dissolution of Eudragit S100 occurs above pH 7.0^37^, therefore it is used for colon specific drug delivery as it hinders drug release in the stomach and small intestine^38^. Thymoquinone (TQ); (2-isopropyl-5-methyl-1,4-benzoquinone)^39^ was employed as a model anticancer drug from a natural source. It is the main ingredient of the volatile oil obtained from *Nigella sativa* (Ranunculaceae) seeds^40,41^. TQ is a lipophilic compound with molecular weight of 164.2^42^, log P of 2.54^43^ and a low aqueous solubility of about 500 μg/ml^44^. TQ possesses antioxidant^45,46^, anti-inflammatory^47,48^, anti-hyperlipidemic^49^, anti-depressant^50^ and antimicrobial activities^51^, and its use in combination with chemotherapeutic agents reduces their side effects^52,53^. TQ possesses antiproliferative activity on various types of cancer including colorectal carcinoma^54^, and was found to induce cell cycle arrest and apoptosis in HCT-116 human colon cancer cells^55^. However, TQ suffers from poor aqueous solubility which limits its applications^56^.

Therefore, encapsulation of TQ in the oily core of polymeric nanocapsules would provide a solution for its poor solubility^57^. AA conjugated to Eudragit S100 would provide targeted system for colon cancer associated with sigma receptors overexpression. Particle size analysis, surface morphology, zeta potential, entrapment efficiency % (EE%), in vitro drug release and physical stability were performed for characterization of nanocapsules. Finally, the study was completed by a cytotoxicity assessment on three colon cancer cell lines; HT-29, HCT-116 and Caco-2 cells. To the best of our knowledge, this study represents the first attempt to use AA as a targeting ligand on the surface of polymeric nanocapsules. It is also the first study in which AA-conjugated Eudragit S100 was synthesized.

## Materials and methods

### Materials

Absolute ethyl alcohol, acetone, ammonia solution (33%), n-butanol, dichloromethane (DCM), glacial acetic acid, methanol, potassium dihydrogen phosphate, silica gel 60–120 mesh for column chromatography (manufactured by Alpha chemika, India), sodium bicarbonate and sodium hydroxide were purchased from El-Nasr Pharmaceutical Co., Cairo, Egypt. *N*-Boc-1,6-hexanediamine, deuterated solvents; chloroform (CDCl_3_) and dimethyl sulfoxide (C_2_D_6_OS) and Span 60 were purchased from Sigma Aldrich Co., Germany. Chloroform was purchased from Honeywell International Inc., Germany. Dialysis tubing cellulose membrane, average flat width 33 mm, 14,000 Da molecular weight cut-off, dimethyl sulfoxide (DMSO), 3-(4,5-dimethylthiazol-2-yl)-2,5-diphenyltetrazolium bromide (MTT) and isopropanol were purchased from Sigma Aldrich Co., USA. *N*,*N′*-Dicyclohexylcarbodiimide (DCC), 4-(dimethylamino) pyridine (DMAP), 4-methoxybenzoic acid and trifluoroacetic acid (TFA) were purchased from Alfa Aesar, USA. Double ring cellulose filter papers 102 Qualitative, medium filter speed (pore 20–30 µm), diameter 9 cm were purchased from Hangzhou Whatman-Xinhua filter paper Co., Ltd, China. Eudragit S100 was provided as a gift from Evonik Industries AG, Germany. Heat-inactivated fetal bovine serum, Roswell Park Memorial Institute medium (RPMI 1640), streptomycin/penicillin and trypsin were purchased from Invitrogen corporation, California, USA. Hydrochloric acid (33%) was purchased from Biochem Chemopharma Co., France. Labrafac PG (propylene glycol dicaprylocaprate) was provided as a gift from Gattefosse' Co., France. Nanosep Centrifugal Devices with Omega Membrane (100 kDa cut-off) were purchased from Pall Corporation, USA. Ninhydrin was purchased from Winlab Co., UK. Thin layer chromatography (TLC) plates (silica gel 60 F254 packed on Aluminum sheets) and Tween 80 were purchased from Merck Co., Germany. Thymoquinone (TQ) was purchased from Frinton laboratories, USA. Uranyl acetate -2- hydrate was purchased from Pratap chemical industries, India.

### Synthesis of AA-conjugated Eudragit S100

#### Synthesis of *tert*-butyl (6-(4-methoxybenzamido)hexyl)carbamate (product I)

4-methoxybenzoic acid (183 mg, 1.2 mmol) was mixed with *N*-Boc-1,6-hexanediamine (210.6 mg, 1 mmol) in DCM (30 ml) in the presence of DCC (207 mg, 1 mmol) and DMAP (24.4 mg, 0.2 mmol), as catalyst, at room temperature for 48 h at an appropriate stirring rate. This was followed by filtration and evaporation of the solvent under reduced pressure using the rotary evaporator; model RVO5, ST (Janke and Kunkel, IKA Laboratories, Staufen, Germany) then the product was left in the laminar flow hood (Flores Valles Co., Madrid, Spain) overnight to get rid of any traces of the organic solvent.The aforementioned synthesis step is designated as step (1) and the product I was obtained.

#### Separation of filtrate products obtained in step (1)

This was done by column chromatography using silica as the stationary phase and DCM/methanol (80:1) as a solvent system. This purification step is designated as step (2). Confirmation of the experiment was done by TLC plate using the developing solvent DCM/methanol (40:1) followed by visualization under U.V. lamp (Vilberlourmat VL-6LC, 6 W–365 nm tube, 6 W–254 nm, France) at 254 nm. Chemical composition of the obtained products was confirmed by ^1^H-NMR (Bruker Avance III HD—400 MHz, Switzerland).

#### Deprotection of the purified product I and formation of product II [synthesis of *N*-(6-aminohexyl)-4-methoxybenzamide (AA conjugate)]

The purified product obtained in step (2) was stirred at room temperature with 50% TFA in DCM for the deprotection of Boc group^58^. The reaction was monitored by the TLC plate until complete consumption of the starting material^59^. The solvent was then evaporated under reduced pressure using the rotary evaporator. The obtained product was then neutralized with distilled water and 5% sodium bicarbonate solution^60^ followed by product extraction with chloroform. The deprotection was confirmed on TLC plate, using the solvent system DCM/methanol (40:1) and visualization with U.V. lamp. Ninhydrin indicator 1.5% was prepared by dissolving 0.75 g ninhydrin in a mixture of 50 ml n-butanol and 1.5 ml glacial acetic acid, followed by sonication for 5 min for further confirmation of the deprotection reaction.

### Synthesis of AA-Eudragit S100 conjugate

A suitable amount of AA conjugate (20 mg, 0.08 mmol) was dissolved in 7 ml distilled acetone and added portion-wise to a solution of Eudragit S100 (158.6 mg, 0.54 mmol methacrylic acid) in 8 ml distilled acetone. This was followed by the addition of DCC (16.4 mg, 0.08 mmol) and DMAP (5.1 mg, 0.042 mmol). One ml methanol was added to the reaction mixture to complete the polymer dissolution. The reaction mixture was left on the magnetic stirrer for 72 h at room temperature, followed by TLC. After filtration of the reaction mixture, the filtrate was placed in a dialysis bag against methanol on a magnetic stirrer at room temperature to get rid of any unreacted molecules. The reaction was monitored using TLC until the complete disappearance of any spot of product II. Finally, the solvent was allowed to evaporate in the laminar flow hood.

### Products characterization

#### ^1^H-NMR

The percent of substitution of carboxylic acid groups in Eudragit S100 by AA was calculated from the integration of the ^1^H-NMR spectrum by comparing the integration of the aromatic protons to that of the rest of the protons in the polymer using the following equations:

1 $${\text{Integration}}\;{\text{of}}\;{\text{aromatic}}\;{\text{protons}}\;{\text{at}}\;\delta \;\left( {6.9{-}8} \right) = {\text{fraction}}\;{\text{of}}\;{\text{AA}}*4\,{\text{H}}$$

2 $${\text{Integration}}\;{\text{of}}\;{\text{aliphatic}}\;{\text{protons}}\;{\text{at}}\;\delta \;\left( {0{-}{4}} \right) = \left[ {\left( {{\text{fraction}}\;{\text{of}}\;{\text{Eudragit}}\;*\;{19}\;{\text{H}}} \right) + \left( {{\text{fraction}}\;{\text{of}}\;{\text{AA}}\;*\;{15}\;{\text{H}}} \right)} \right]$$

3 $$\begin{aligned} &\% \;{\text{of}}\;{\text{substitution}}\;{\text{of}}\;{\text{carboxylic}}\;{\text{acid}}\;{\text{groups}}\;{\text{in}}\;{\text{Eudragit}}\,{\text{S1}}00\;{\text{by}}\;{\text{AA}} \\&\quad= [{\text{fraction}}\;{\text{of}}\;{\text{AA}}/\left( {{\text{fraction}}\;{\text{of}}\;{\text{AA}} + {\text{fraction}}\;{\text{of}}\;{\text{Eudragit}}} \right)]*{1}00 \end{aligned}$$

Cho et al.^61^ used the integration in ^1^H-NMR to calculate the degree of substitution of PEG in hyaluronic acid-ceramide polymer. It should be pointed out that in the interpretation of ^1^H-NMR spectra, splitting of the peaks will be referred to using the following letters; (s) for singlet, (d) for duplet and (m) for multiplet peaks.

For ^1^H-NMR measurements, the sample dissolved in 0.2 ml of the deuterated solvent was placed in 3 mm NMR tube and measured using Bruker Avance III HD—400 MHz. TopSpin 3.2 NMR processing software was used for NMR data analysis and the acquisition and processing of NMR spectra.

^1^H-NMR measurement of product I was carried out under the following conditions; [solvent: deuterated chloroform CDCl_3_, number of scans (NS): 32]. ^1^H-NMR measurements of unmodified Eudragit S100 and AA-conjugated Eudragit S100 were carried out under the following conditions; [solvent: deuterated DMSO, NS: 128] and [solvent: deuterated DMSO, NS:256] respectively.

#### Mass spectrometry

Mass spectrometry was carried out using mass spectrometer (GC-2010 Shimadzu, Japan), under the following conditions; ionization mode: electron impact, ion source temperature: 250 °C, start m/z: 50, end m/z: 500, and electron voltage: 70 eV. ChemBioDraw Ultra (version 13) was used to draw the chemical structures and to aid in elucidating the structures of different compounds.

### Preparation of TQ-loaded nanocapsules

Non-conjugated TQ-loaded nanocapsules were prepared using the nanoprecipitation method described by Fessi et al.^57,62^. Following preformulation studies using various oils and surfactants, the lipophilic solution consisted of 0.2 ml oil (Labrafac PG), 62.5 mg polymer (Eudragit S100), 48 mg surfactant (Span 60) and 25 mg TQ dissolved in acetone and sonicated. The organic phase was added to an aqueous solution containing surfactant (Tween 80) and mixed using a magnetic stirrer. The solvent was removed by evaporation under ambient conditions while being stirred overnight^63^. AA-targeted TQ-loaded nanocapsules were also prepared using the nanoprecipitation method following the same procedure previously described except that the lipophilic solution consisted of 0.2 ml Labrafac PG, 62.5 mg AA-conjugated Eudragit S100, 48 mg surfactant (Span 60) and 25 mg TQ dissolved in organic solvent (acetone and methanol mixture).

### Characterization of TQ-loaded nanocapsules

The particle size, PDI and zeta potential of the nanocapsules were measured using the Malvern Zetasizer nano ZS (Malvern Instruments Ltd, Malvern, UK)^64^ at a scattering angle of 173°. A sample of 200 μl of the nanocapsules dispersion was diluted with deionized water and the particle size, PDI and zeta potential were measured at 25 °C.

TEM was used to determine the morphology of the nanocapsules formulations. A sample of the nanocapsules dispersion was appropriately diluted with deionized water. It was then deposited on a carbon coated copper grid, left to dry and then negatively stained with a super-saturated solution of uranyl acetate followed by microscopic examination (Jeol—JEM—1010, operating at 80 kV, Japan).

The concentration of the unentrapped drug was determined by centrifugation of 600 μl of the nanocapsules dispersion inside the nanosep at 4 °C and 7,000 rpm for one hour, followed by another centrifugation cycle at 4 °C and 8,000 rpm for one hour using the cooling centrifuge (Hermle Labortechnik GmBH, model Z216MK, Germany). After centrifugation, the concentration of the unentrapped drug in the filtrate was measured spectrophotometrically (Jenway UV–visible spectrophotometer, Staffordshire, UK) at 253 nm after appropriate dilution with absolute ethanol. EE% of TQ in the nanocapsules was calculated using the following equation:

4 $${\text{EE}}\% = \frac{{[{\text{Total}}\;{\text{drug}} - {\text{Free}}\;{\text{drug}}]}}{{{\text{Total}}\;{\text{drug}}}} \times {1}00$$

### In vitro release of TQ from nanocapsules

Free TQ was removed from the nanocapsules dispersion using the exhaustive dialysis method^65^ using a cellulose membrane (Dialysis tubing cellulose membrane 14,000 Da molecular weight cut-off) bag against 200 ml double-distilled water on a magnetic stirrer at room temperature, until complete removal of free drug from the nanocapsules dispersion. After separating the free TQ from the drug-loaded nanocapsules, the membrane diffusion technique using the modified USP apparatus I was used to evaluate the drug release from the loaded nanocapsules. The cellulose membrane was soaked before use in double-distilled water at room temperature overnight. Three ml of the nanocapsules formulation containing an amount of 2.7 mg TQ were placed in a glass cylinder having a length of 5 cm and diameter of 2 cm. This cylinder was covered at one end with previously soaked cellulose membrane and fixed to the apparatus shaft. The cylinder was placed in the dissolution vessel of the USP dissolution tester (Pharma test, type PTW-2, Hainburg, Germany) containing 100 ml of the medium with pH 1.2 (0.1 N HCl). The apparatus was adjusted to a constant speed (50 rpm) and a temperature of 37 ± 0.5 °C. At predetermined time intervals (1, 2, 3, 4, 5, 6, 7, 8 and 24 h), 1 ml samples were withdrawn and assayed spectrophotometrically at 258 nm for the drug content. Each withdrawn sample was replaced by an equal volume of the medium of a certain pH. A pH shift was carried out; after 2 h from the beginning of the experiment, the pH of the release medium was changed to 5.5 by addition of a certain volume of alkaline phosphate buffer (composed of 2.5 M potassium dihydrogen phosphate containing 16.72% (w/v) sodium hydroxide as described by Kondo et al.^66^). Using different amounts of alkaline phosphate buffer, the pH of the dissolution medium was raised to pH 6.8 and 7.4 at the 4th and 6th hours respectively. The added volumes of the buffer were taken into consideration during calculation of the percentage of drug released at each time interval. The pH of the release medium was adjusted by adding a certain volume of the alkaline phosphate buffer till the pH-meter (Jenway, Model 3510, UK) read the desired pH value.

### Determination of the physical stability of nanocapsules

The nanocapsules dispersions were stored at 2–8 °C, and samples were withdrawn at different time intervals; 45 days and 90 days^57^. These samples were appropriately diluted with deionized water and examined for particle size and zeta potential. EE% was also measured after 45 and 90 days following the same procedure described previously for EE% determination of the freshly-prepared formulation.

### MTT cytotoxicity assay

Different human colon cancer cell lines (HCT-116, HT-29 and Caco-2) were grown in the tissue culture lab of Egyptian Company for Vaccines and Sera (Vacsera, Giza, Egypt). At the time of the experiment, the cells were checked for viability and confluency before being grown on the appropriate growth medium, RPMI 1640, supplemented with 1% mixture of 100 mg/ml of streptomycin, 100 units/ml of penicillin and 10% of heat-inactivated fetal bovine serum in a humidified, 5% (V/V) CO_2_ atmosphere at 37 °C.

Exponentially growing cells from different cancer cell lines were trypsinized, counted and seeded at the appropriate densities (2000–10,000 cells/0.33 cm^2^ well) into 96-well microtiter plates. Cells were then incubated in a humidified atmosphere at 37 °C for 24 h. Afterwards, cells were exposed to a solution of TQ in DMSO or to dialyzed dispersions of TQ-loaded nanocapsules at different concentrations for 24 or 48 h. The cytotoxicity of each concentration was assessed in triplicate. The viability of treated cells was determined using MTT technique as follows: the media were removed; cells were incubated with 200 μl of 5% MTT solution/well and were allowed to metabolize the dye into colored-insoluble formazan crystals for 2 h. The remaining MTT solution was discarded from the wells and the formazan crystals were dissolved in 200 µl/well acidified isopropanol for 30 min, covered with aluminum foil and shaken using a MaxQ 2000 plate shaker (Thermo Fisher Scientific Inc., Mississauga, Canada) at room temperature. Absorbance readings were measured at 570 nm using a Stat Fax 4,200 plate reader (Awareness Technology, Inc., Florida, USA). The cell viability was expressed as the percentage of treated cells relative to untreated control cells and the concentration that induces 50% of maximum inhibition of cell proliferation (IC_50_) was determined using GraphPad Prism version 5 software. Blank formulations (prepared without TQ) were also assessed for their cytotoxic potential^67^.

### Statistical analysis

Results were expressed as mean ± standard deviation (SD). Statistical data analysis was conducted using GraphPad InStat software. A comparison of the data was performed using either student’s *t* test or one way ANOVA.

## Results and discussion

### Synthesis of AA-conjugated Eudragit S100

**Figure 1:**
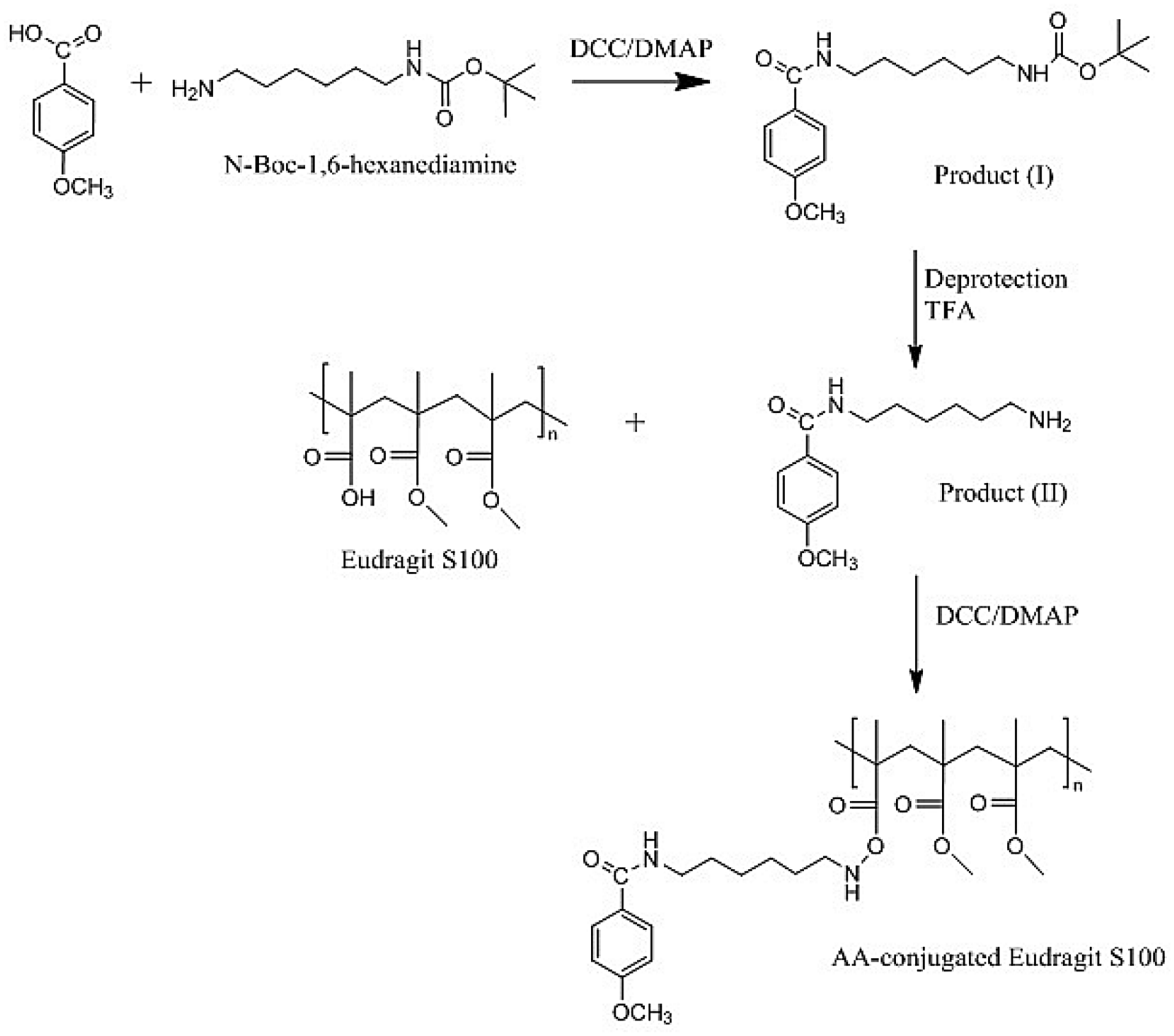
Synthesis of AA-conjugated Eudragit S100.

AA-Eudragit S100 polymer conjugate was synthesized via three successive steps; (1) conjugation of 4-methoxybenzoic acid to *N*-Boc-1,6-hexanediamine via DCC/DMAP coupling^68,69^ [DCC; *N*,*N′*-Dicyclohexylcarbodiimide, DMAP; 4-(dimethylamino) pyridine] to prepare *tert*-butyl (6-(4-methoxybenzamido)hexyl) carbamate (product I), (2) deprotection to remove the Boc group of product I by trifluoroacetic acid (TFA) to prepare *N*-(6-aminohexyl)-4-methoxybenzamide (product II), and (3) conjugation of the deprotected product II primary amine to Eudragit S100 free carboxylic groups as illustrated in Fig. 1.

### Product I, *tert*-butyl (6-(4-methoxybenzamido)hexyl) carbamate

**Figure 2:**
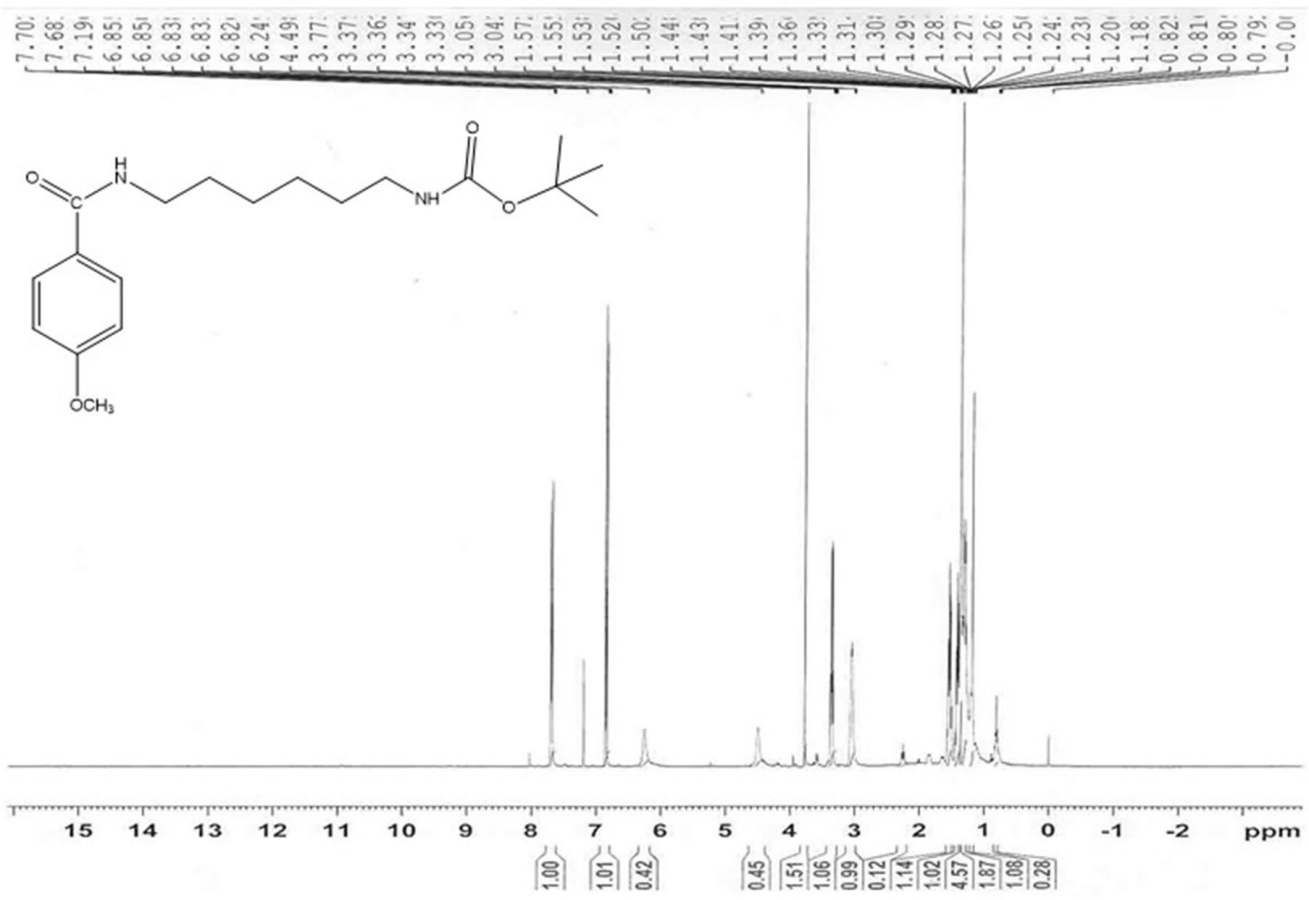
^1^H-NMR of product (I).

During the preparation of product I, insoluble *N*,*N*′-dicyclohexylurea byproduct of the DCC/DMAP coupling reaction was removed by filtration. Product I was purified from *N*-acyl urea derivative by silica gel column chromatography using the solvent system dichloromethane (DCM)/methanol (80:1), with a yield of 36.67% for product I and a retention factor (R_f_) value of 0.545 [thin layer chromatography (TLC), mobile phase is DCM/methanol 40:1]. The chemical composition of product Iwas confirmed by ^1^H-NMR (Fig. 2).

Product I: ^1^H-NMR: Chemical shift(δ) 6.83, 6.85 (2H, d) of the 2 aromatic CH ortho to the methoxy group, 7.68, 7.7 (2H, d) of the 2 aromatic CH meta to the methoxy group, 4.49 (1H, s) of the NH attached to the Boc group, 6.24 (1H, s) of the NH group of the amide bond attached to methoxybenzene, 3.77 (3H, s) of CH_3_ of the methoxy group, 3.33–3.37 (2H, m) of the CH_2_ alpha to the NH group of the amide bond attached to methoxybenzene, 3.04–3.05 (2H, m) of the CH_2_ alpha to the NH attached to the Boc group, 1.5–1.57 (4H, m) of the 2 CH_2_ beta to the NH attached to the Boc group and beta to the NH group of the amide bond attached to methoxybenzene, 1.39–1.44 (4H, m) of the 2 CH_2_ gamma to the NH attached to the Boc group and gamma to the NH group of the amide bond attached to methoxybenzene,1.36 (9H, s) of the 3 CH_3_ of the Boc group.

**Figure 3:**
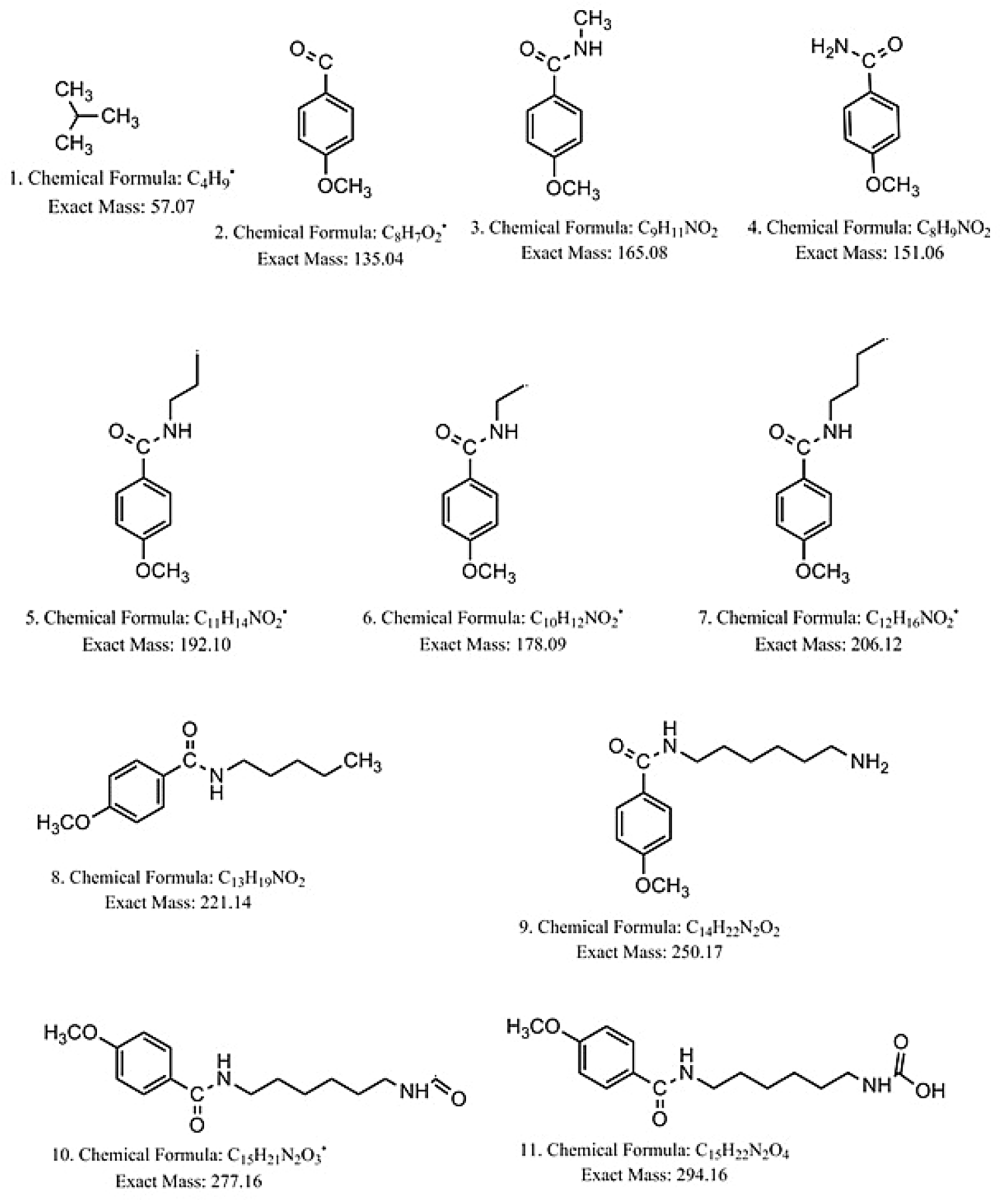
Suggested compounds obtained by fragmentation of product (I).

The formation of product I was confirmed by mass spectrometry together with its fragmentation products as shown in Supplementary information 1 and Fig. 3. The appearance of a peak with m/z = 350.2 confirms the formation of the product as the molecular weight of the product is 350.22.

### Product II, *N*-(6-aminohexyl)-4-methoxybenzamide

The deprotection reaction of product I by TFA was monitored by TLC. In addition to detection under U.V., ninhydrin indicator was used to detect the free primary amine group of product II. The spot of the protected product (product I) disappeared totally after one hour of the reaction with TFA in DCM and a new spot for the deprotected product appeared at the baseline. The obtained product was extracted with chloroform after neutralization with aqueous sodium bicarbonate solution^60^ to liberate the unionized free amine group from its TFA salt. The higher polarity of product II, with a free primary amine group relative to its protected form (product I) resulted in an R_f_ of zero with the solvent system DCM/methanol (40:1). In addition, product II appeared as a purple spot after staining the TLC plate with ninhydrin, indicating the presence of free primary amino group and hence successful deprotection of product I^70^. No purple spot appeared for the Boc protected product I. The yield of product II was 94.5%.

### AA-Eudragit S100 conjugate

Figure 1 illustrates the reaction between AA conjugate and Eudragit S100 in the presence of DCC and DMAP. The success of the coupling reaction was confirmed using the TLC plate and DCM/methanol/ammonia (6:2:0.15) as solvent. A dark spot appeared under U.V. at the baseline for the modified polymer (R_f_ of zero) and no dark spot appeared for the unmodified Eudragit. Polymer purification was conducted by dialysis against methanol to remove unconjugated product II, as well as any other small molecules byproducts. The purification was confirmed by TLC usingninhydrin indicator, where a purple spot was evident only for the free AA conjugate (R_f_ = 0.125) using the solvent system DCM/methanol/ammonia (6:2:0.15).

**Figure 4:**
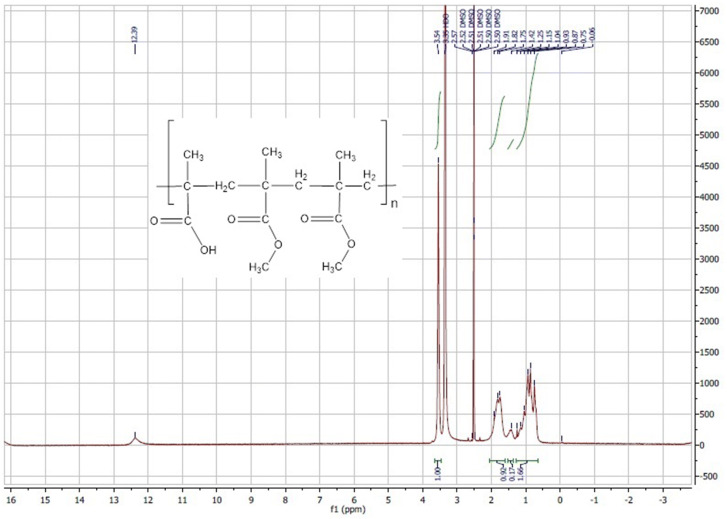
^1^H-NMR of unmodified Eudragit S100.

**Figure 5:**
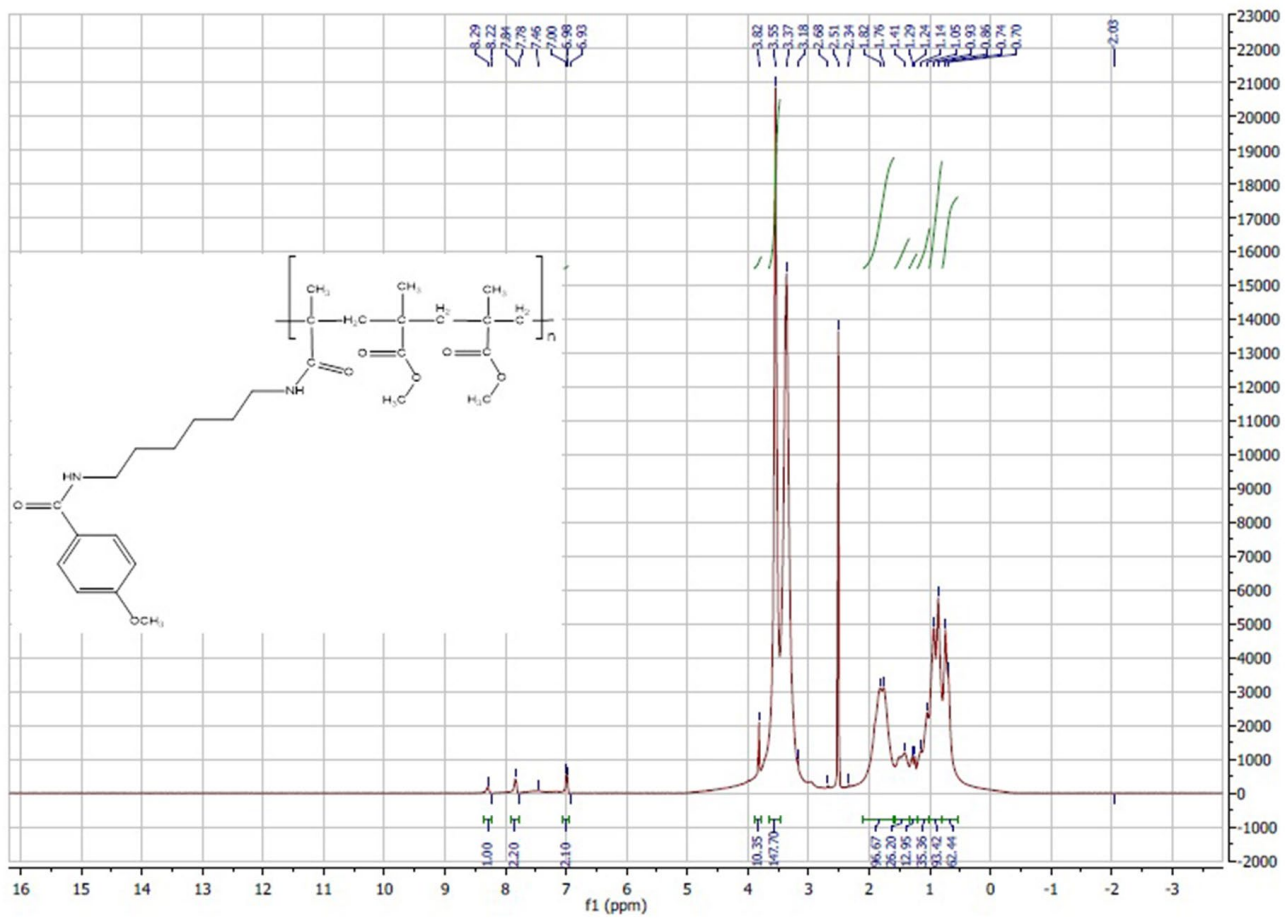
^1^H-NMR of AA-conjugated Eudragit S100.

The unmodified Eudragit S100 ^1^H-NMR shows δ 12.39 (1H, s) of the carboxylic acid, 3.54 (3H, s) of the methyl group in the ester bond of methylmethacrylate unit of the polymer, 1.75–1.91(2H) of the CH_2_ group linking methylmethacrylate and methacrylic acid, 1.25 (6H) of the 2 CH_3_ groups where each of them is beta to either methacrylic acid or methacrylate (Fig. 4). On the other hand, the AA-conjugated Eudragit S100 ^1^H-NMR showed that in addition to the same peaks of the unmodified polymer (0.65–1.9 ppm), peaks at δ 6.93, 6.98 (2H, d) and δ 7.78, 7.84 (2H, d) were identified (Fig. 5). Those peaks appearing in the range of δ 6.93–7.84 represent the two groups of aromatic protons in AA and confirmed AA-polymer conjugation, since any unconjugated deprotected product II was removed by the dialysis step. This result concurred with that obtained by Yang et al.^71^, in which the aromatic protons of AA appeared at δ 6.97 and 7.83. The average percent of substitution of carboxylic acid groups in Eudragit S100 by AA was around 6.87% as calculated using the integration of the ^1^H-NMR, by substitution in the equations mentioned in the methods in the experimental section.

### Characterization of TQ-loaded nanocapsules

The particle size of the prepared nanocapsules ranged from 217 to 231.5 nm with a PDI from 0.229 to 0.306 (less than 0.4) (Table 1). A size above 200 nm was reported to be necessary to avoid diffusion through the intestinal mucus and consequent cellular uptake^72^. Subudhi et al.^38^ prepared oral Eudragit S100-coated citrus pectin nanoparticles loaded with 5-fluorouracil with an average particle size of 218.12 ± 10.25 nm for colorectal cancer. In addition, Khatik et al.^73^ prepared oral curcumin-loaded Eudragit S100-coated chitosan nanoparticles with average particle size of 236 ± 3.2 nm as a colon-specific delivery in Wistar rats. Furthermore, Schaffazick et al.^74^ prepared melatonin-loaded EudragitS100 nanocapsules using the nanoprecipitation technique of 236 ± 20 nm in diameter.

**Table 1:** Particle size, PDI, zeta potential and entrapment efficiency % of the prepared nanocapsular formulations.

Formula code	Type of oil	Type of polymer	Particle size mean ± S.D (nm)	PDI mean ± S.D	Zeta potential mean ± S.D. (mV)	EE% mean ± S.D
F-P	Labrafac	Eudragit S100	231.5 ± 5.2	0.306 ± 0.028	− 36.3 ± 0.9	85.7 ± 5
F-AA	Labrafac	Anisamide-conjugated Eudragit S100	217.2 ± 5.6	0.229 ± 0.011	− 38.97 ± 1.3	90.5 ± 0.55

Each formula contained 25 mg TQ.

**Figure 6:**
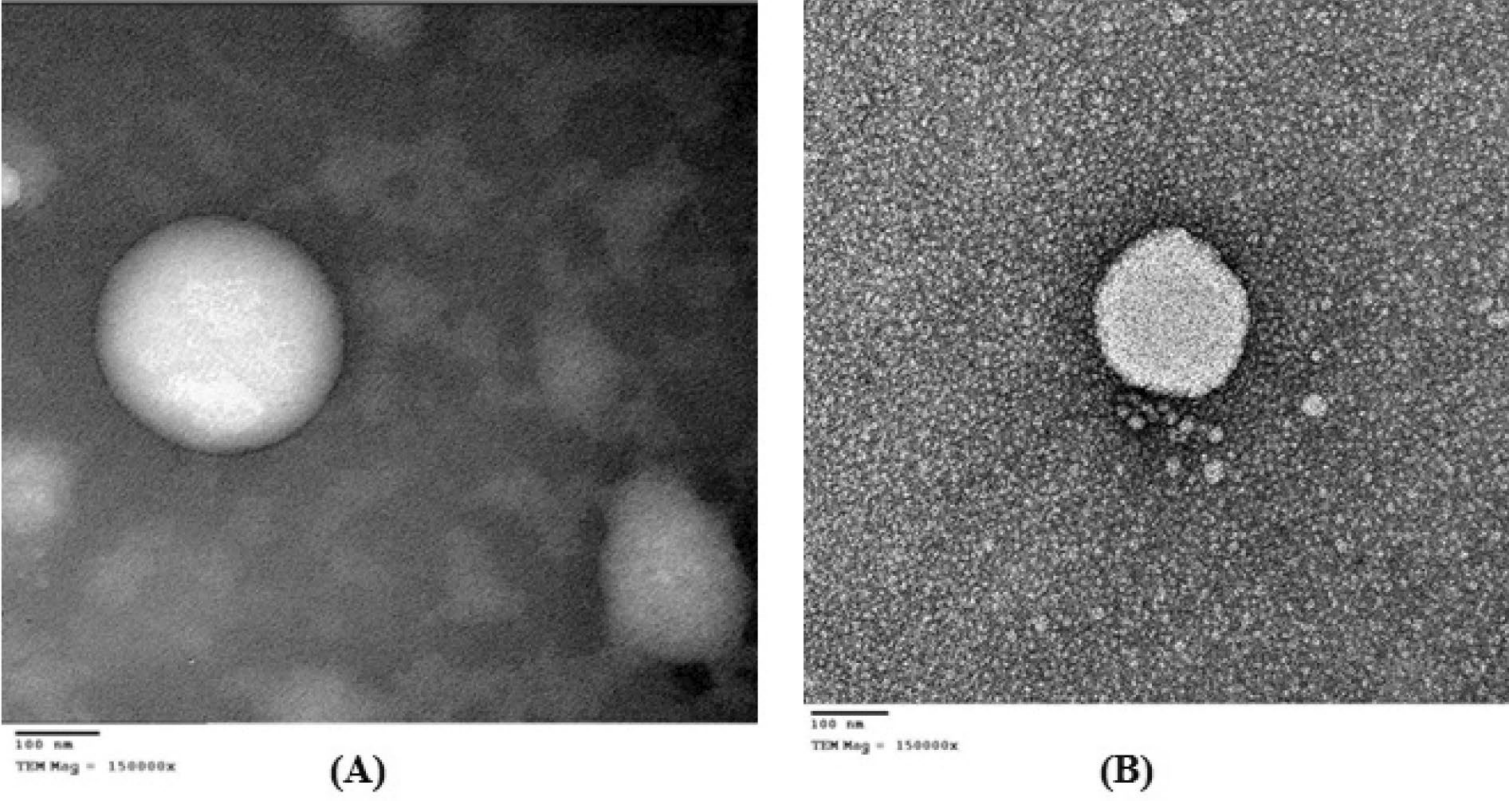
TEM photomicrographs of TQ-loaded nanocapsules (F-P) at a magnification of × 150,000 (**A**) and AA-conjugated TQ-loaded nanocapsules (F-AA) at a magnification of × 150,000 (**B**).

The transmission electron microscopy (TEM) photomicrographs showed the formation of spherical shaped or nearly spherical nanocapsules. Similar to, Fessi et al.^62^ and Tagliari et al.^75^, the photomicrographs revealed the presence of an opaque film or coat surrounding the oily core for unconjugated (Fig. 6A), and conjugated Eudragit S100 nanocapsules (Fig. 6B).

Table 1 also discloses zeta potential values of − 36 to − 39 mV due to the presence of free carboxylic acid groups of methacrylic acid^38^, similar to what was reported by Schaffazick et al.^76^ and Coco et al.^77^ using the same polymer. A zeta potential higher than − 30 mV, is expected to prevent particles aggregation^78,79^.

Finally a high EE% of TQ ranging from 86 to 90.5% was also obtained (Table 1). This can be attributed to the good lipid solubility of TQ^65^ with a log *P* = 2.54^43^. In a likewise manner, the EE% of gemcitabine derivatives inside polycyanoacrylate nanocapsules was affected by the extent of lipophilicity of derivatives^80^.

### In vitro release of TQ from nanocapsules

**Figure 7:**
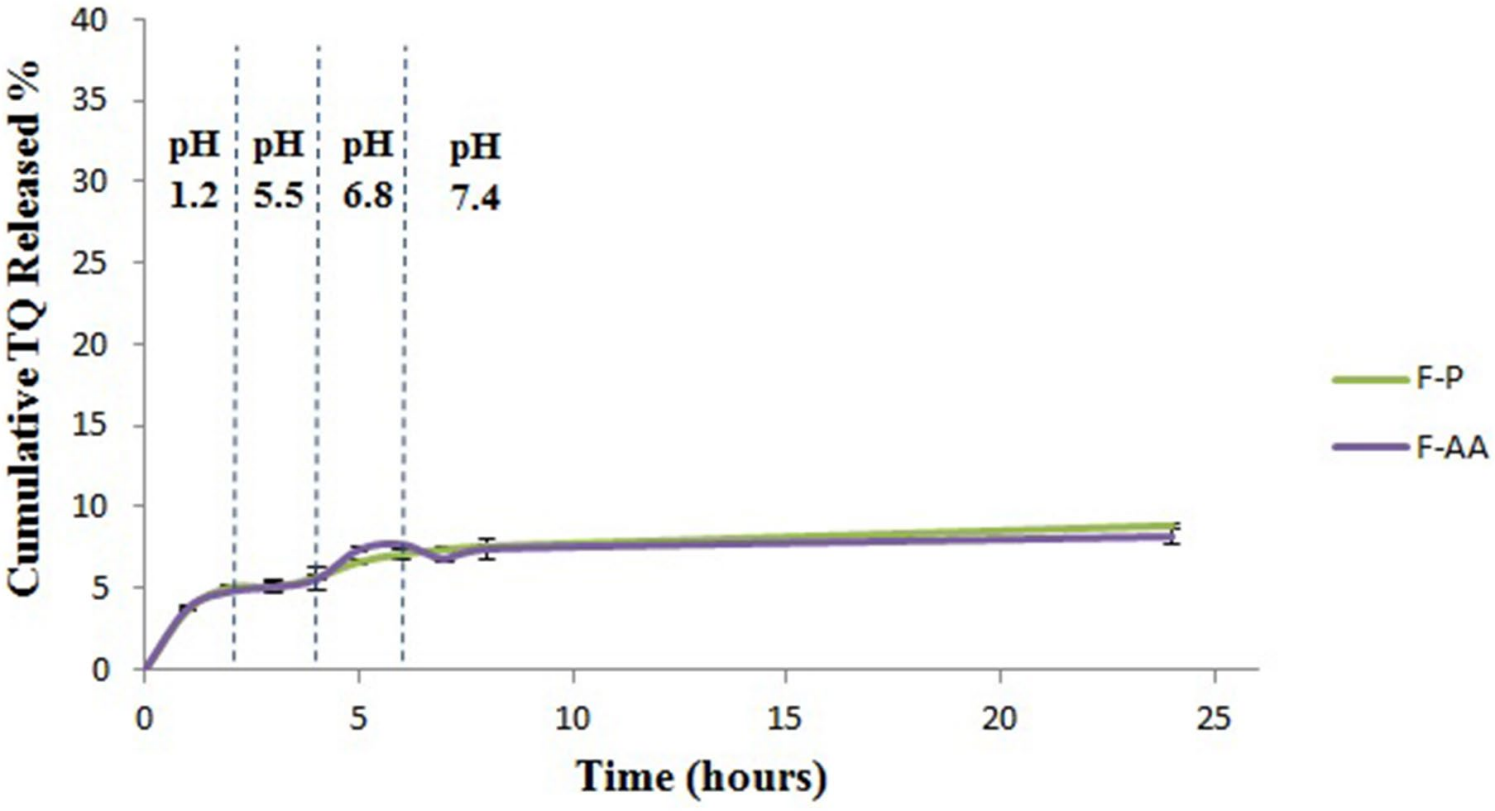
Release profiles of TQ from different nanocapsular formulations at different pHs.

The cumulative % released of TQ after 24 h was 8.865 and 8.196 for F-P and F-AA respectively. These results (Fig. 7) came in accordance with the results obtained by Odeh et al.^65^ who reported a minimal drug release from TQ liposomes. Since Eudragit S100 is a pH sensitive polymer, we hypothesized that the release of TQ from nanocapsules would be pH-dependent. However owing to the lipophilicity of TQ (log *P* = 2.54), it actually had strong affinity to the oily phase of the nanocapsules (Labrafac PG) that even after the polymer shell dissolved by the increase in pH the released amount of TQ was still low, with no significant difference from other pHs (*P* > 0.05). A drug liberation, limited to the tumor inside-post-uptake would increase local drug concentration as previously described^81^.

### Physical stability of TQ nanocapsules

No significant change in particle size, PDI, zeta potential or EE% of the prepared nanocapsules after storage for 45 days and 90 days at 2–8 °C (*P* > 0.05) was evident in Table 2.

**Table 2:** Physical stability of nanocapsular formulations after 45 and 90 days at 2–8 °C.

Formula	Parameter	Initial	After 45 days	After 90 days
F-P	Particle size (nm)	231.5 ± 5.2	227.8 ± 12.6	220.4 ± 13.9
	PDI	0.306 ± 0.028	0.317 ± 0.049	0.3 ± 0.058
	Zeta potential (mV)	− 36.35 ± 0.9	− 33.25 ± 0.2	− 35.65 ± 0.3
	EE%	88.6 ± 2.8	91.1 ± 1.7	92.8 ± 3.8
F-AA	Particle size (nm)	217.2 ± 5.6	211.8 ± 6	223.7 ± 19.9
	PDI	0.229 ± 0.011	0.223 ± 0.016	0.263 ± 0.042
	Zeta potential (mV)	− 38.97 ± 1.3	− 40.3 ± 0.8	− 39.1 ± 2.1
	EE%	90.5 ± 0.55	90.09 ± 1.2	90.08 ± 2.65

### In vitro cytotoxicity of TQ-loaded nanocapsules

The cytotoxicity of different TQ formulations was tested by MTT (3-[4,5-dimethylthiazol-2-yl]-2,5-diphenyltetrazolium bromide) assay in three different human colon cancer cell lines; HT-29, HCT-116 and Caco-2 cells after 24 and 48 h of incubation. HT-29 and HCT-116 are derived from colorectal primary carcinoma of different stages, while Caco-2 cells are derived from non-specific colon cancer cells^82^. The tested formulations were: free TQ dissolved in dimethyl sulfoxide (DMSO), TQ-loaded Eudragit S100 nanocapsules (F-P), blank Eudragit S100 nanocapsules (B-P), TQ-loaded AA-conjugated Eudragit S100 nanocapsules (F-AA) and blank AA-conjugated Eudragit S100 nanocapsules (B-AA).

**Figure 8:**
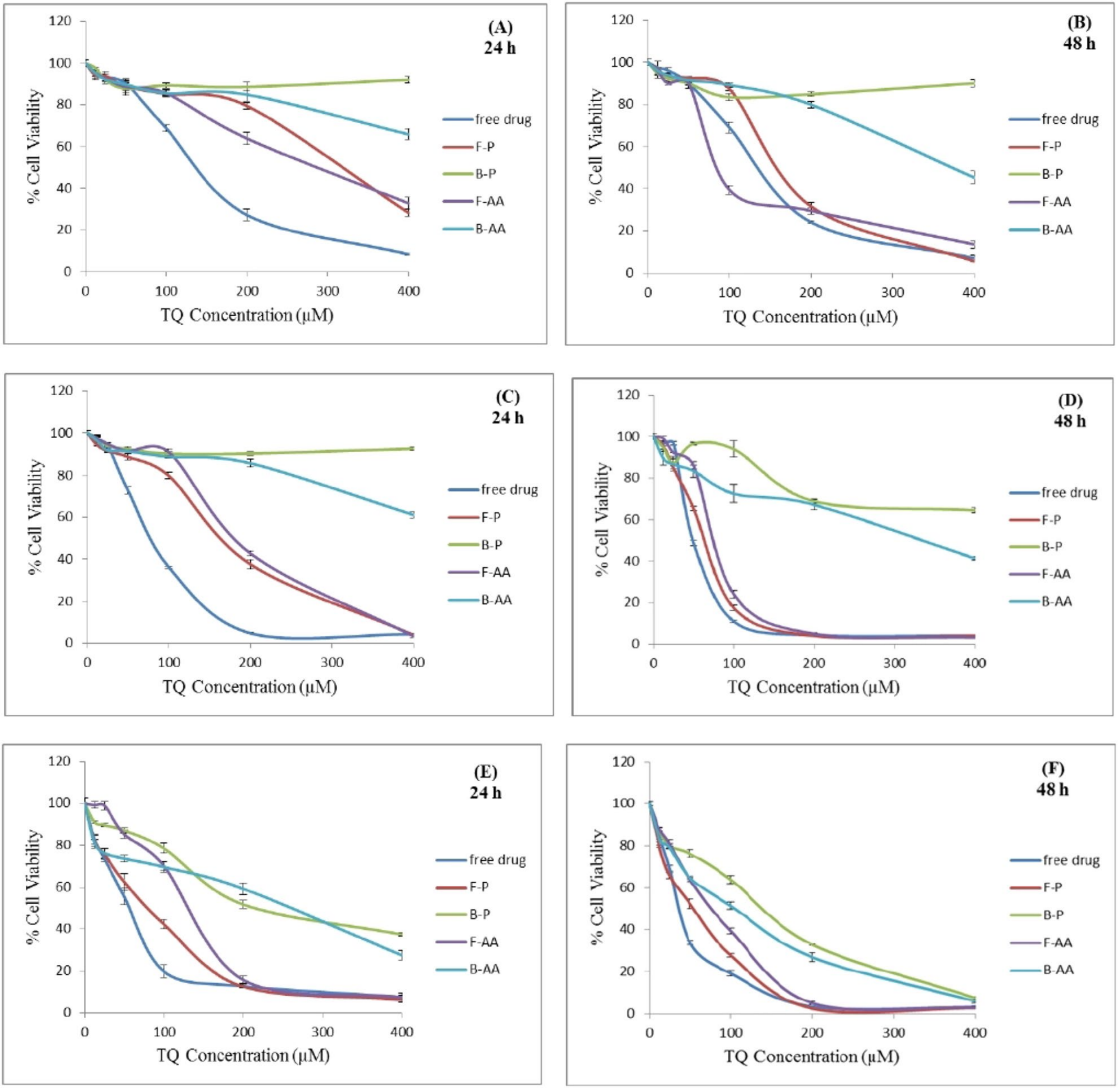
Cytotoxicity of free TQ and various nanoformulations on HT-29 cells (**A**, **B**), HCT-116 cells (**C**, **D**) and Caco-2 cells (**E**, **F**) after incubation for 24 and 48 h respectively.

Upon testing the cytotoxicity on HT-29 cells, results revealed the safety of blank nanocapsules with a high cell viability, except for the highest nanoparticles concentration of (B-AA) probably due to increased blocking of the sigma receptors^83,84^ (Fig. 8A). After 24 h incubation period, free TQ was significantly more cytotoxic than both targeted and non-targeted nanocapsules against HT-29 cells (*P* < 0.05). The respective IC_50_ values were: 144.3 µM ± 5.7, 287.7 µM ± 12.7 and 313.7 µM ± 5.5. These results were in accordance with those obtained by Zheng et al.^85^ where the IC_50_ value of free drug was significantly lower than those of non-targeted and transferrin-targeted nanoparticles, and may be attributed to the controlled drug release as shown in the in vitro release experiment. Significantly lower IC_50_ of F-AA than F-P(*P* < 0.05) may be attributed to the higher uptake of AA-targeted nanocapsules by the sigma receptor-overexpressing HT-29 cells^86^.

However at 48 h and illustrated in Fig. 8B, the respective IC_50_ values were: 143.3 µM ± 2.5, 89.7 µM ± 0.9 and 167 µM ± 1.7 confirming the superior cytotoxicity of F-AA. The obtained value was close to that obtained by El-Najjar et al.^87^, reporting 110 µM as the IC_50_ for TQ. The constant cytotoxicity of free TQ on HT-29 cells during 48 h may be attributed to the resistance of HT-29 cells to TQ. While studying the antiproliferative effect of TQ on the colon cancer cell lines: HT-29, HCT-116, DLD-1, LoVo, and Caco-2 cells, the previous authors observed that HT-29 was the most resistant cell line to the growth suppression effect mediated by TQ. They explained that HT-29 cell line displays elevated levels of DT-diaphorase (DTD) enzyme^88^. This enzyme catalyzes the conversion of quinones to hydroquinones via two-electron reduction thus preventing the generation of reactive oxygen species (ROS)^89^. An enzymatically-induced reduction, which can be regarded as a detoxification process^90^ might have induced TQ resistance^87^.

The higher cytotoxicity of F-AA than free TQ against HT-29 cells after 48 h may be explained by the enhanced uptake of these TQ nanocapsules by binding to the sigma receptors, which are also overexpressed on the surface of HT-29 cells. Besides, the presence of TQ inside the nanocapsules helped the drug to escape the detoxification process by unknown mechanism, and the anticancer activity of released TQ occurred differently. No more ROS generation but rather a cell cycle arrest^55^ or induction of apoptosis might have occurred^55,91^. Park et al.^91^ revealed that TQ exerts its anticancer activity via induction of apoptosis and ROS generation in human renal carcinoma Caki cells. TQ induced apoptosis in these cells by downregulating the expression of the anti-apoptotic proteins c-FLIP and Bcl-2. Bcl-2 downregulation caused by TQ, also contributed to mitochondrial death by inducing loss of mitochondrial membrane potential.

The targeting properties might have changed the pharmacokinetics of the drug inside the cell, improving its anticancer activity. F-P showed a 47% decrease in IC_50_ by time, while the change mounted 69% in case of the actively-targeted nanoparticles.

As for HT-29 cells, the B-AA and B-P nanocapsules did not affect cell viability of HCT-116 cells significantly except at the highest particulate concentration of B-AA after 24 h (Fig. 8C). However, both of them caused a decrease in cell viability after 48 h, but with the effect of B-AA being more pronounced (Fig. 8D). It can be observed that the cytotoxicity of the free drug increased to a lesser extent after 48 h compared to targeted and non-targeted nanocapsules and its IC_50_ was reduced by only 38.5% after 48 h. On the other hand, both types of nanocapsules showed about 60% decrease in their IC_50_ by prolonging the incubation time from 24 to 48 h, confirming once more an enhanced cell uptake of drug nanoparticles. AA-targeted nanocapsules did not show superior cytotoxicity over non-targeted ones against HCT-116 cells owing to a low expression of sigma receptors. HCT-116 cells lack sigma-2 receptors and express a lower level of sigma-1 receptors than HT-29 cells^86^ and AA specificity towards sigma-1 or sigma-2 receptors is still unclear and requires further investigations^33^.

Finally in Caco-2 cells, B-AA and B-P showed a decrease in cell viability after 24 h increasing in effect upon extending the time to 48 h as illustrated in Fig. 8E, F. In addition, this cell line was unable to discriminate between different formulations in cytotoxicity at high drug concentrations and especially after 48 h. The cell viabilities were also of lower values than with the other two cell lines. This may be attributed to the lower expression of cytochrome P450 in Caco-2 cells^92^. Furthermore, Caco-2 cells lack mucus secretion^93^, which functions as a physical barrier providing protection against foreign particles and toxins^94^.

To sum up, in order of decreased expression of sigma receptors, the cell lines could be arranged in the following order: HT-29 cells > Caco-2 cells > HCT-116 cells^86^. AA specificity towards sigma-1 or sigma-2 receptors is still unclear and requires further investigations^33^. Moreover, HT-29 cell line displays an elevated level of DTD enzyme, while HCT-116 cells show lower expression of the gene that codes for DTD compared to HT-29 cells and Caco-2 cells totally lack DTD enzyme^88^. The IC_50_ values of free TQ on HT-29, HCT-116 and Caco-2 cells after incubation for 48 h were: 143.3, 49.7 and 38.1 µM respectively. The results of our study concur with the difference in the expression level of DTD enzyme between the three cell lines. They also concurred with those obtained by El-Najjaret al.^74^ in which HT-29 cells exhibited the highest resistance to TQ (IC_50_ = 110 µM). The cytotoxicity of TQ-loaded nanocapsules on the three cell lines was time-dependent. Table 3 summarizes the IC_50_ values of the tested TQ formulations on different colon cancer cell lines after different incubation periods.

**Table 3:** IC_50_ (µM) of free TQ and TQ-loaded nanoformulations on colon cancer cell lines after incubation for 24 and 48 h.

Cell line type	Free TQ		F-P		F-AA	
	24 h	48 h	24 h	48 h	24 h	48 h
HT-29	144.3 ± 5.7	143.3 ± 2.5	313.7 ± 5.5	167 ± 1.7	287.7 ± 12.7	89.7 ± 0.9
HCT-116	80.8 ± 1.6	49.7 ± 1.9	170.3 ± 3.2	66.4 ± 1	184.3 ± 1.5	78.1 ± 1
Caco-2	58 ± 4.8	38.1 ± 1	79.7 ± 5.8	54.2 ± 3.3	136 ± 4.3	78.1 ± 1

Results of the present study reveal the promising targeting potential of TQ loaded AA-targeted nanocapsules for sigma receptors overexpressed in colon cancer cells.

## Conclusions

In this study, conjugated and non-conjugated polymeric nanocapsules loaded with TQ were prepared for targeting colon cancer using Eudragit S100 as a pH-sensitive polymer and AA as a ligand for sigma receptors generally overexpressed by colon cancer cells. AA-targeted nanocapsules exhibited higher cytotoxicity than non-conjugated ones and free TQ against HT-29 cells after 48 h of incubation. This can be attributed to the elevated level of expression of sigma receptors on HT-29 cells resulting in enhanced uptake of nanocapsules. This was also confirmed by the resistance of this cell line to the free drug. Therefore, improving treatment efficiency and reducing the incidence of adverse effects and drug resistance could be expected by these nanocapsules. In order to further prove the targeting ability of the presented nanocapsules, futuristic in vivo experiments should be conducted in order to evaluate the biodistribution of the nanocapsules, as well as their therapeutic potential.

## Supplementary information

Supplementary file1 (DOCX 87 kb)

## References

[1] Ashford ME (2014). Synthesis and in vitro evaluation of tetrahydroisoquinolines with pendent aromatics as sigma-2 (sigma2) selective ligands. Org. Biomol. Chem..

[2] Hellewell SB (1994). Rat liver and kidney contain high densities of σ1 and σ2 receptors: characterization by ligand binding and photoaffinity labeling. Eur. J. Pharmacol..

[3] Hellewell SB, Bowen WD (1990). A sigma-like binding site in rat pheochromocytoma (PC12) cells: decreased affinity for (+)-benzomorphans and lower molecular weight suggest a different sigma receptor form from that of guinea pig brain. Brain Res..

[4] Cobos EJ, Entrena JM, Nieto FR, Cendan CM, Del Pozo E (2008). Pharmacology and therapeutic potential of sigma(1) receptor ligands. Curr. Neuropharmacol..

[5] Fontanilla D (2009). The hallucinogen N,N-dimethyltryptamine (DMT) is an endogenous sigma-1 receptor regulator. Science.

[6] Rousseaux CG, Greene SF (2015). Sigma receptors [sigmaRs]: biology in normal and diseased states. J. Recept. Signal Transduct. Res..

[7] Tesei A (2019). Anti-tumor efficacy assessment of the sigma receptor pan modulator RC-106. A promising therapeutic tool for pancreatic cancer. Front. Pharmacol..

[8] Nguyen L (2015). Role of sigma-1 receptors in neurodegenerative diseases. J. Pharmacol. Sci..

[9] Sun YT (2018). Synthesis and pharmacological evaluation of 6,7-dimethoxy-1,2,3,4-tetrahydroisoquinoline derivatives as sigma-2 receptor ligands. Eur. J. Med. Chem..

[10] Aydar E, Palmer CP, Djamgoz MB (2004). Sigma receptors and cancer: possible involvement of ion channels. Cancer Res..

[11] Thomas GE (1990). Sigma and opioid receptors in human brain tumors. Life Sci..

[12] John CS, Bowen WD, Varma VM, McAfee JG, Moody TW (1995). Sigma receptors are expressed in human non-small cell lung carcinoma. Life Sci..

[13] Moody TW, Leyton J, John C (2000). Sigma ligands inhibit the growth of small cell lung cancer cells. Life Sci..

[14] Crawford KW, Bowen WD (2002). Sigma-2 receptor agonists activate a novel apoptotic pathway and potentiate antineoplastic drugs in breast tumor cell lines. Cancer Res..

[15] Vilner BJ, John CS, Bowen WD (1995). Sigma-1 and sigma-2 receptors are expressed in a wide variety of human and rodent tumor cell lines. Cancer Res..

[16] Bem WT (1991). Overexpression of sigma receptors in nonneural human tumors. Cancer Res..

[17] Penke B, Fulop L, Szucs M, Frecska E (2018). The role of sigma-1 receptor, an intracellular chaperone in neurodegenerative diseases. Curr. Neuropharmacol..

[18] Nicholson HE (2019). Divergent cytotoxic and metabolically stimulative functions of sigma-2 receptors: structure-activity relationships of 6-acetyl-3-(4-(4-(4-fluorophenyl)piperazin-1-yl)butyl)benzo[d]oxazol-2(3H)-one (SN79) derivatives. J. Pharmacol. Exp. Ther..

[19] Shoghi KI (2013). Quantitative receptor-based imaging of tumor proliferation with the sigma-2 ligand [(18)F]ISO-1. PLoS ONE.

[20] Caveliers V, Everaert H, John CS, Lahoutte T, Bossuyt A (2002). Sigma receptor scintigraphy with N-[2-(1'-piperidinyl)ethyl]-3-(123)I-iodo-4-methoxybenzamide of patients with suspected primary breast cancer: first clinical results. J. Nucl. Med..

[21] John CS, Vilner BJ, Geyer BC, Moody T, Bowen WD (1999). Targeting sigma receptor-binding benzamides as in vivo diagnostic and therapeutic agents for human prostate tumors. Cancer Res..

[22] Yang D (2017). Design and investigation of a [(18)F]-labeled benzamide derivative as a high affinity dual sigma receptor subtype radioligand for prostate tumor imaging. Mol. Pharm..

[23] Banerjee R, Tyagi P, Li S, Huang L (2004). Anisamide-targeted stealth liposomes: a potent carrier for targeting doxorubicin to human prostate cancer cells. Int. J. Cancer.

[24] Li SD, Huang L (2006). Targeted delivery of antisense oligodeoxynucleotide and small interference RNA into lung cancer cells. Mol. Pharm..

[25] Nakagawa O, Ming X, Huang L, Juliano RL (2010). Targeted intracellular delivery of antisense oligonucleotides via conjugation with small-molecule ligands. J. Am. Chem. Soc..

[26] Ramzy L, Nasr M, Metwally AA, Awad GAS (2017). Cancer nanotheranostics: a review of the role of conjugated ligands for overexpressed receptors. Eur. J. Pharm. Sci..

[27] Chen Y, Bathula SR, Yang Q, Huang L (2010). Targeted nanoparticles deliver siRNA to melanoma. J. Invest. Dermatol..

[28] Yang Y, Li J, Liu F, Huang L (2012). Systemic delivery of siRNA via LCP nanoparticle efficiently inhibits lung metastasis. Mol. Ther..

[29] Chen WH, Lecaros RL, Tseng YC, Huang L, Hsu YC (2015). Nanoparticle delivery of HIF1alpha siRNA combined with photodynamic therapy as a potential treatment strategy for head-and-neck cancer. Cancer Lett..

[30] Kim SK, Foote MB, Huang L (2013). Targeted delivery of EV peptide to tumor cell cytoplasm using lipid coated calcium carbonate nanoparticles. Cancer Lett..

[31] Garg NK, Dwivedi P, Campbell C, Tyagi RK (2012). Site specific/targeted delivery of gemcitabine through anisamide anchored chitosan/poly ethylene glycol nanoparticles: an improved understanding of lung cancer therapeutic intervention. Eur. J. Pharm. Sci..

[32] Fitzgerald KA (2016). A novel, anisamide-targeted cyclodextrin nanoformulation for siRNA delivery to prostate cancer cells expressing the sigma-1 receptor. Int. J. Pharm..

[33] Evans JC (2017). Formulation and evaluation of anisamide-targeted amphiphilic cyclodextrin nanoparticles to promote therapeutic gene silencing in a 3D prostate cancer bone metastases model. Mol. Pharm..

[34] Urandur S (2018). Anisamide-anchored lyotropic nano-liquid crystalline particles with aie effect: a smart optical beacon for tumor imaging and therapy. ACS Appl. Mater. Interfaces.

[35] Luan X (2019). Anisamide-targeted PEGylated gold nanoparticles designed to target prostate cancer mediate: enhanced systemic exposure of siRNA, tumour growth suppression and a synergistic therapeutic response in combination with paclitaxel in mice. Eur. J. Pharm. Biopharm..

[36] Jahangir MA, Khan R, Sarim Imam S (2018). Formulation of sitagliptin-loaded oral polymeric nano scaffold: process parameters evaluation and enhanced anti-diabetic performance. Artif. Cells Nanomed. Biotechnol..

[37] Yoo J-W, Giri N, Lee CH (2011). pH-sensitive Eudragit nanoparticles for mucosal drug delivery. Int. J. Pharm..

[38] Subudhi MB (2015). Eudragit S100 coated citrus pectin nanoparticles for colon targeting of 5-Fluorouracil. Materials.

[39] Salim LZ (2013). Thymoquinone induces mitochondria-mediated apoptosis in acute lymphoblastic leukaemia in vitro. Molecules.

[40] Gali-Muhtasib H, Roessner A, Schneider-Stock R (2006). Thymoquinone: a promising anti-cancer drug from natural sources. Int. J. Biochem. Cell Biol..

[41] Woo CC, Kumar AP, Sethi G, Tan KHB (2012). Thymoquinone: potential cure for inflammatory disorders and cancer. Biochem. Pharmacol..

[42] Ng WK (2015). Thymoquinone-loaded nanostructured lipid carrier exhibited cytotoxicity towards breast cancer cell lines (MDA-MB-231 and MCF-7) and cervical cancer cell lines (HeLa and SiHa). Biomed Res. Int..

[43] Singh A, Ahmad I, Akhter S, Ahmad MZ, Iqbal Z (2012). Thymoquinone: major molecular targets, prominent pharmacological actions and drug delivery concerns. Curr. Bioact. Compd..

[44] Salmani JM, Asghar S, Lv H, Zhou J (2014). Aqueous solubility and degradation kinetics of the phytochemical anticancer thymoquinone; probing the effects of solvents, pH and light. Molecules.

[45] Nagi MN, Mansour MA (2000). Protective effect of thymoquinone against doxorubicin-induced cardiotoxicity in rats: a possible mechanism of protection. Pharmacol. Res..

[46] Safhi MM (2016). Neuromodulatory effects of thymoquinone in extenuating oxidative stress in chlorpromazine treated rats. Acta Pol. Pharm..

[47] Boudiaf K (2016). Thymoquinone strongly inhibits fMLF-induced neutrophil functions and exhibits anti-inflammatory properties in vivo. Biochem. Pharmacol..

[48] Vaillancourt F (2011). Elucidation of molecular mechanisms underlying the protective effects of thymoquinone against rheumatoid arthritis. J. Cell. Biochem..

[49] Fakhria A, Gilani SJ, Imam SS (2019). Formulation of thymoquinone loaded chitosan nano vesicles: in-vitro evaluation and in-vivo anti-hyperlipidemic assessment. J. Drug Deliv. Sci. Technol..

[50] Alam M (2018). Formulation and evaluation of nano lipid formulation containing CNS acting drug: molecular docking, in-vitro assessment and bioactivity detail in rats. Artif. Cells Nanomed. Biotechnol..

[51] Jrah Harzallah H, Kouidhi B, Flamini G, Bakhrouf A, Mahjoub T (2011). Chemical composition, antimicrobial potential against cariogenic bacteria and cytotoxic activity of Tunisian *Nigella sativa* essential oil and thymoquinone. Food Chem..

[52] Sarman H, Bayram R, Benek SB (2016). Anticancer drugs with chemotherapeutic interactions with thymoquinone in osteosarcoma cells. Eur. Rev. Med. Pharmacol. Sci..

[53] Cascella M (2017). Role of *Nigella sativa* and its constituent Thymoquinone on chemotherapy-induced nephrotoxicity: evidences from experimental animal studies. Nutrients.

[54] Gali-Muhtasib H (2008). Thymoquinone reduces mouse colon tumor cell invasion and inhibits tumor growth in murine colon cancer models. J. Cell. Mol. Med..

[55] Gali-Muhtasib H (2004). Thymoquinone extracted from black seed triggers apoptotic cell death in human colorectal cancer cells via a p53-dependent mechanism. Int. J. Oncol..

[56] Singh A (2013). Nanocarrier based formulation of Thymoquinone improves oral delivery: stability assessment, in vitro and in vivo studies. Colloids Surf. B Biointerfaces.

[57] El-Gogary RI (2014). Polyethylene glycol conjugated polymeric nanocapsules for targeted delivery of quercetin to folate-expressing cancer cells in vitro and in vivo. ACS Nano.

[58] Park J, Lee HY, Cho M-H, Park SB (2007). Development of a Cy3-labeled glucose bioprobe and its application in bioimaging and screening for anticancer agents. Angew. Chem. Int. Ed. Engl..

[59] Hartwig S, Nguyen MM, Hecht S (2010). Exponential growth of functional poly(glutamic acid) dendrimers with varying stereochemistry. Polym. Chem..

[60] Johnson KT, Gribb TE, Smoak EM, Banerjee IA (2010). Self-assembled nanofibers from leucine derived amphiphiles as nanoreactors for growth of ZnO nanoparticles. Chem. Commun..

[61] Cho H-J (2012). Polyethylene glycol-conjugated hyaluronic acid-ceramide self-assembled nanoparticles for targeted delivery of doxorubicin. Biomaterials.

[62] Fessi H, Puisieux F, Devissaguet JP, Ammoury N, Benita S (1989). Nanocapsule formation by interfacial polymer deposition following solvent displacement. Int. J. Pharm..

[63] Govender T, Stolnik S, Garnett MC, Illum L, Davis SS (1999). PLGA nanoparticles prepared by nanoprecipitation: drug loading and release studies of a water soluble drug. J. Control. Release.

[64] Chauhan N (2017). Development of chitosan nanocapsules for the controlled release of hexaconazole. Int. J. Biol. Macromol..

[65] Odeh F, Ismail SI, Abu-Dahab R, Mahmoud IS, Al Bawab A (2012). Thymoquinone in liposomes: a study of loading efficiency and biological activity towards breast cancer. Drug Deliv..

[66] Kondo N (1994). Improved oral absorption of enteric coprecipitates of a poorly soluble drug. J. Pharm. Sci..

[67] Said-Elbahr R, Nasr M, Alhnan MA, Taha I, Sammour O (2016). Nebulizable colloidal nanoparticles co-encapsulating a COX-2 inhibitor and a herbal compound for treatment of lung cancer. Eur. J. Pharm. Biopharm..

[68] Neises B, Steglich W (1978). Simple method for the esterification of carboxylic acids. Angew. Chem. Int. Ed..

[69] Han S-Y, Kim Y-A (2004). Recent development of peptide coupling reagents in organic synthesis. Tetrahedron.

[70] Schetz JA, Anderson PAV (1993). Investigations of lipid components of neurone-enriched membranes of the jellyfish *Cyanea capillata*. J. Exp. Biol..

[71] Yang W (2016). Efficient and targeted suppression of human lung tumor xenografts in mice with methotrexate sodium encapsulated in all-function-in-one chimeric polymersomes. Adv. Mater..

[72] Plapied L, Duhem N, des Rieux A, Préat V (2011). Fate of polymeric nanocarriers for oral drug delivery. Curr. Opin. Colloid Interface Sci..

[73] Khatik R (2013). Colon-specific delivery of curcumin by exploiting Eudragit-decorated chitosan nanoparticles in vitro and in vivo. J. Nanopart. Res..

[74] Schaffazick SR, Pohlmann AR, Mezzaliraa G, Guterres SS (2006). Development of nanocapsule suspensions and nanocapsule spray-dried powders containing melatonin. J. Braz. Chem. Soc..

[75] Tagliari MP (2015). Development of oral nifedipine-loaded polymeric nanocapsules: physicochemical characterisation, photostability studies, in vitro and in vivo evaluation. Quim. Nova.

[76] Schaffazick SR (2008). Incorporation in polymeric nanocapsules improves the antioxidant effect of melatonin against lipid peroxidation in mice brain and liver. Eur. J. Pharm. Biopharm..

[77] Coco R (2013). Drug delivery to inflamed colon by nanoparticles: comparison of different strategies. Int. J. Pharm..

[78] Singh R, Lillard JW (2009). Nanoparticle-based targeted drug delivery. Exp. Mol. Pathol..

[79] Nayak D, Ashe S, Rauta PR, Kumari M, Nayak B (2016). Bark extract mediated green synthesis of silver nanoparticles: evaluation of antimicrobial activity and antiproliferative response against osteosarcoma. Mater. Sci. Eng. C Mater. Biol. Appl..

[80] Stella B (2007). Encapsulation of gemcitabine lipophilic derivatives into polycyanoacrylate nanospheres and nanocapsules. Int. J. Pharm..

[81] Arpicco S, Milla P, Stella B, Dosio F (2014). Hyaluronic acid conjugates as vectors for the active targeting of drugs, genes and nanocomposites in cancer treatment. Molecules.

[82] Ahmed D (2013). Epigenetic and genetic features of 24 colon cancer cell lines. Oncogenesis.

[83] Brent PJ, Pang GT (1995). Sigma binding site ligands inhibit cell proliferation in mammary and colon carcinoma cell lines and melanoma cells in culture. Eur. J. Pharmacol..

[84] Vilner BJ, de Costa BR, Bowen WD (1995). Cytotoxic effects of sigma ligands: sigma receptor-mediated alterations in cellular morphology and viability. J. Neurosci..

[85] Zheng Y (2010). Transferrin-conjugated lipid-coated PLGA nanoparticles for targeted delivery of aromatase inhibitor 7α-APTADD to breast cancer cells. Int. J. Pharm..

[86] Safrany, S. T., Abbas, H., Ferry, D. R. & Brimson, J. M. Sigma receptor content in a range of cancer cell lines. In *PB147 (poster session), Pharmacology 2014, British Pharmacological Society, Queen Elizabeth II Conference Centre, London, UK* (2014).

[87] El-Najjar N (2010). Reactive oxygen species mediate thymoquinone-induced apoptosis and activate ERK and JNK signaling. Apoptosis.

[88] Traver RD (1992). NAD(P)H: quinone oxidoreductase gene expression in human colon carcinoma cells: characterization of a mutation which modulates DT-diaphorase activity and mitomycin sensitivity. Cancer Res..

[89] Cullen JJ (2003). Dicumarol inhibition of NADPH: quinone oxidoreductase induces growth inhibition of pancreatic cancer via a superoxide-mediated mechanism. Cancer Res..

[90] Karczewski JM, Peters JG, Noordhoek J (1999). Quinone toxicity in DT-diaphorase-efficient and -deficient colon carcinoma cell lines. Biochem. Pharmacol..

[91] Park EJ, Chauhan AK, Min KJ, Park DC, Kwon TK (2016). Thymoquinone induces apoptosis through downregulation of c-FLIP and Bcl-2 in renal carcinoma Caki cells. Oncol. Rep..

[92] van Breemen RB, Li Y (2005). Caco-2 cell permeability assays to measure drug absorption. Expert Opin. Drug Metab. Toxicol..

[93] Pan F, Han L, Zhang Y, Yu Y, Liu J (2015). Optimization of Caco-2 and HT29 co-culture in vitro cell models for permeability studies. Int. J. Food Sci. Nutr..

[94] Johansson ME (2008). The inner of the two Muc2 mucin-dependent mucus layers in colon is devoid of bacteria. Proc. Natl. Acad. Sci. U. S. A..

