# Supplementary material for: Novel thymoquinone lipidic core nanocapsules with anisamide-polymethacrylate shell for colon cancer cells overexpressing sigma receptors
Source: Sci Rep. 2020 Jul 3;10:10987. doi: 10.1038/s41598-020-67748-2 (PMC7335198; doi:10.1038/s41598-020-67748-2)
Supplement: Supplementary file 1 — Supplementary file1 (DOCX 87 kb) [file 41598_2020_67748_MOESM1_ESM.docx]

**Novel thymoquinonelipidic core nanocapsules with anisamide-polymethacrylate shell for colon cancer cells overexpressing sigma receptors**

**Lydia Ramzy^1^, AbdelkaderA. Metwally^1,2^, Maha Nasr^1^*, Gehanne A.S. Awad^1^**

*^1^Department of Pharmaceutics and Industrial Pharmacy, Faculty of Pharmacy, Ain Shams University, Cairo, Egypt*

*^2^Department of Pharmaceutics, Faculty of Pharmacy, Health Sciences Center, Kuwait University, Kuwait.*

**Supplementary 1**

**Product I:**

Mass spectrometry: The absorption intensities of compound (1) (Mwt = 57.07), compound (2) (Mwt =135.04), compound (3) (Mwt =165.08), compound (4) (Mwt =151.06), compound (5) (Mwt =192.1), compound (6) (Mwt =178.09), compound (7) (Mwt =206.12), compound (8) (Mwt =221.14), compound (9) (Mwt =250.17), compound (10) (Mwt =277.16) and compound (11) (Mwt =294.16) were relatively high (295865, 353828,51372, 28902, 19375,24462, 10370, 40942, 16667, 36805 and 26199) respectively and the peaks of these compounds confirmed the formation of the amide bond by nucleophilic attack.

**
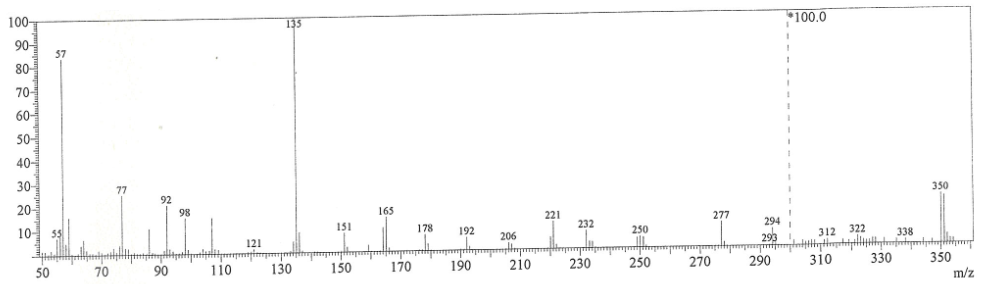
**

**Mass spectrum of product (I).**
